# Is it possible to align the teaching of radiology in undergraduate medicine with that employed in graduate and continuing medical education courses?

**DOI:** 10.1590/0100-3984.2017.0094

**Published:** 2018

**Authors:** Alexandre Ferreira da Silva

**Affiliations:** 1 Famaz - Faculdade Metropolitana da Amazônia, Belém, PA, Brazil.

Dear Editor,

The correlation among clinical, radiology, and pathology is considered one of the most important elements of the medical science teaching/learning process, being widely used in the training of specialists not only in radiology and diagnostic imaging but also in several other fields of medicine; it also represents a basic tool for the construction of strategies aimed at continuing medical education, using the principles of interdisciplinarity and transdisciplinarity in education^(^^1^^)^.

The ongoing changes in Brazilian medical education have created opportunities to redirect and rescale radiology instruction in undergraduate education, as well as in clinical and surgical specialization courses, with an impact on the training and continuing education of radiologists. This process has been influenced by studies demonstrating that shifting the focus of the teaching/learning process from the teacher to the students, together with the use of active learning methodologies and teaching techniques that favor the development of reasoning for problem-solving at all stages of medical education, has advantages for the restructuring of curricula and learning objectives, as well as for changing the mentality of instructors^(^^1^^-^^7^^)^.

In undergraduate education, breaking down disciplinary walls allows the contents to be integrated, thus promoting the mobilization of knowledge and facilitating the understanding of physiopathology and radiological signs in each clinical context; however, most Brazilian medical schools still use the disciplinary structure, misaligned in relation to the integrative and multidimensional pedagogical strategies practiced by postgraduate programs where in-service teaching bridges the gap between theory and practice in health care provision, education, and management^(^^1^^,^^2^^)^.

Although the correlation among clinical, radiology and pathology plays a central role in the teaching of diagnostic imaging at all stages of medical education, other relevant elements should be included in undergraduate and graduate education. Among those elements, the appropriate choice of complementary tests has become a critical point of medical training, because the poor use of diagnostic methods has a strong negative impact on patient care and places a burden on the health care system, whether public or private. It is important to develop an advantageous teaching strategy as a useful tool to counteract those inadequacies^(^^1^^,^^2^^,^^4^^,^^6^^)^.

Therefore, it is possible to align the teaching of radiology to undergraduate medical students with that used in graduate and continuing medical education courses, anchoring the three stages in the correlation among clinical, radiology, and pathology. However, in the undergraduate stage, it is fundamental to adopt integrated curricula, regardless of the methodologies employed. It is also essential that questions regarding the rational use of diagnostic tools, including the appropriate way of requesting examinations as well as how to understand the text of the radiology report, should be continually examined, because progressive technological changes imply the adoption of new linguistic terms and procedures, which require periodic updating.
